# EEG Window Length Evaluation for the Detection of Alzheimer’s Disease over Different Brain Regions

**DOI:** 10.3390/brainsci9040081

**Published:** 2019-04-14

**Authors:** Katerina D. Tzimourta, Nikolaos Giannakeas, Alexandros T. Tzallas, Loukas G. Astrakas, Theodora Afrantou, Panagiotis Ioannidis, Nikolaos Grigoriadis, Pantelis Angelidis, Dimitrios G. Tsalikakis, Markos G. Tsipouras

**Affiliations:** 1 Department of Medical Physics, Medical School, University of Ioannina, GR45110 Ioannina, Greece; ktzimourta@cc.uoi.gr (K.D.T.); astrakas@uoi.gr (L.G.A.); 2 Department of Informatics and Telecommunications, School of Informatics and Telecommunications, University of Ioannina, GR47100 Arta, Greece; tzallas@uoi.gr (N.G.); giannakeas@uoi.gr (A.T.T.); 3 2nd Department of Neurology, AHEPA University Hospital, Aristotle University of Thessaloniki, GR54636 Thessaloniki, Greece; afrantou@gmail.com (T.A.); ioannidispanosgr@yahoo.gr (P.I.); grigoria@med.auth.gr (N.G.); 4 Department of Informatics and Telecommunications Engineering, University of Western Macedonia, GR50100 Kozani, Greece; paggelidis@uowm.gr (P.A.); dtsalikakis@uowm.gr (D.G.T.)

**Keywords:** Alzheimer’s Disease, EEG, detection, mild, moderate, dementia, classification, Random Forests, window length

## Abstract

Alzheimer’s Disease (*AD*) is a neurogenerative disorder and the most common type of dementia with a rapidly increasing world prevalence. In this paper, the ability of several statistical and spectral features to detect *AD* from electroencephalographic (*EEG*) recordings is evaluated. For this purpose, clinical *EEG* recordings from 14 patients with *AD* (8 with mild *AD* and 6 with moderate *AD*) and 10 healthy, age-matched individuals are analyzed. The *EEG* signals are initially segmented in nonoverlapping epochs of different lengths ranging from 5 s to 12 s. Then, a group of statistical and spectral features calculated for each *EEG* rhythm (δ, θ, α, β, and γ) are extracted, forming the feature vector that trained and tested a Random Forests classifier. Six classification problems are addressed, including the discrimination from whole-brain dynamics and separately from specific brain regions in order to highlight any alterations of the cortical regions. The results indicated a high accuracy ranging from 88.79% to 96.78% for whole-brain classification. Also, the classification accuracy was higher at the posterior and central regions than at the frontal area and the right side of temporal lobe for all classification problems.

## 1. Introduction

Alzheimer’s Disease (*AD*) is neurogenerative disease of unknown etiology with a great prevalence in western countries [1]. Patients with *AD* are characterized with a loss of memory, sleeping problems, mood disorders, and general confusion, which are caused by structural irregularities or damage in the synaptic connections, due to amyloid-β plaques and neurofibrillary tangles [2]. In a recent Alzheimer’s report of 2018 [3], the worldwide *AD* prevalence was about 33 million patients out of 50 million people suffering from dementia, making *AD* the most common type of dementia.

A variety of diagnostics procedures are performed to evaluate the cognitive and neuropsychological state of patients with dementia, including neuronal and physical examination, brain imaging, and electroencephalographic (*EEG*) recording. The Mini-Mental State Examination (*MMSE*) [4] and the Clinical Dementia Rating (*CDR*) [5] score are a 30-point scale and a 5-point scale respectively, which are utilized by neurologists to evaluate the cognitive decline and functional performance of patients with *AD*. Higher values of the *CDR* score indicate a more severe condition, whereas higher values of the *MMSE* score shows very mild dementia and a healthy condition (*MMSE* above 28).

An analysis of the *EEG* recordings in *AD* patients is of significant importance, since information of the brain dynamics may shed light on the exact mechanisms of *AD* [6]. Research studies in *AD* over the past 40 years have indicated the alterations in *EEG* complexity, synchrony, and brain dynamics (the slowing of alpha rhythm and the diffuse dominance of theta or delta rhythm) [7]. Several studies have been proposed aimed at finding a correlation between the *MMSE* score and *EEG* features [7,8,9] or discriminating *AD* patients from patients with other neurological conditions through their *EEG* findings. In particular, methods have been proposed for the automated discrimination of *AD* patients from healthy elderly subjects [10,11,12,13,14,15,16], frontotemporal dementia [17], vascular dementia [18], Mild Cognitive Impairment (*MCI*) [19,20], or even epilepsy [21]. Generally, the *EEG* activity is analyzed from each electrode site [6,22] or from electrode clusters [7,8,23]. Studies concerning the structural and functional asymmetry have reported that an early onset of AD affects different lobes [24]. Thus, an analysis of the *EEG* based on electrode clusters that depict different cortical regions may reveal anatomical deficits or differences in the neuronal connection due to other mechanisms [8].

Concerning *AD* detection from *EEG* findings, researchers have suggested several different features, which represent *EEG* complexity, synchrony, and regularity. Relative band power [12,20,25], absolute band power [18], Lempel–Ziv complexity [12,20], Permutation entropy [10,22], Sample entropy [17,22], Spectral entropy [11,15,17,26], Fuzzy entropy [20], automutual information [17], mean frequency [17,27] amplitude modulation [10], central tendency [17], mean [12], variance [12], and zero-crossing [12] are the most frequently extracted *EEG* features for *AD* detection. The features are extracted directly from raw *EEG* segments [10,12,15,19,20] or after a signal decomposition with a Wavelet Analysis [25,27], Power Spectral Density using Berg’s method [28], Hilbert–Huang Transform [10], or Multivariate Multiscale Analysis [11]. Concerning the epoch duration in which the signal is segmented, there is no common agreement regarding the appropriate window length and there is a diversity among research studies [10,11,12,13,14,15,16,17,18,19,20,21,22,24,29]. According to the literature, the *EEG* window length is usually selected between 5 s to 12 s arbitrarily or based on literature survey.

In this study, a method for automated detection of Alzheimer’s Disease is proposed. *EEG* recordings from *AD* patients with moderate and mild *AD* are analyzed along with the *EEG* data from healthy, age-matched individuals in epochs of different length (ranging from 5 to 12 s). The features from both the time and frequency domains are extracted, forming the feature vector to train several classifiers. The evaluation of the window length shows that epochs of 12 s with Random Forests indicate the best classification performance for six classification problems and 5 different brain regions of interest. To the best of our knowledge, this is the first comprehensive study examining a variety of features over multiple window lengths and showing a high classification accuracy. The results of the methodology are presented below.

The paper is organized as follows: In Section 2, the methodology and the extracted linear and nonlinear *EEG* features are addressed. Section 3 presents the obtained results for six classification problems, and Section 4 discusses the obtained results compared to literature findings. Finally, in Section 5, the conclusion and the future directions of this study are presented.

## 2. Materials and Methods

The proposed *AD* detection method consists of three stages. The *EEG* signals acquired from 10 healthy individuals and 14 *AD* patients were initially segmented in nonoverlapping epochs of 8 different lengths. Then, 8 time-based and 30 spectral features were extracted from the *EEG* segments forming the feature vector. Finally, the resulting feature vector was used as input to train a Random Forests classifier.

### 2.1. Data Acquisition

The *EEG* signals used in the methodology were obtained from 24 subjects: 14 patients with *AD* and 10 age-matched, healthy individuals that formed the group “controls”. The evaluation of *AD* severity was performed with the international *MMSE* score and the *CDR* by an experienced neurologist. Thus, 8 out of 14 patients suffered from mild *AD* (*MMSE* scores 19–23), whereas 6 patients suffered from moderate *AD* (*MMSE* scores 10–18). Table 1 presents the demographic characteristics of the participants in the study in terms of the median value and *IQR* (Q1–Q3) range. A pairwise statistical significance analysis between the three groups (controls, mild *AD*, and moderate *AD*) was performed concerning age and the MMSE score. Thus, a Welch ANOVA (*p-value = 0.124,* F(2,21) = 2.473) was performed in order to show that there was not statistically significant difference between the 3 groups with respect to age. Also, concerning the MMSE score that was not normally distributed, a nonparametric Kruskal–Wallis test ($\chi^{2}=21.913$, *p*-value < 0.001, with a mean rank MMSE of 19.5 for the controls, 10.5 for mild *AD*, and 3.5 for moderate *AD*) was performed, aimed at proving that the groups were significantly different in terms of the MMSE score. The statistical analysis was performed using the IBM SPSS Statistics [29].

The recordings were performed at the 2nd Department of Neurology of AHEPA General Hospital of Thessaloniki with the Nihon Kohden EEG 2100 device. The 19 electrodes (Fp1, Fp2, F7, F3, Fz, F4, F8, T3, C3, Cz, C4, T4, T5, P3, Pz, P4, T6, O1, and O2) were placed on the scalp according to the 10–20 International Reference System, and the 2 electrodes (A1 and A2) were placed on the subject’s earlobes (left and right, respectively) for a skin impedance check. Also, the electrodes were placed around the eyes to capture the electrooculogram (*EOG*). The recordings were performed with a bipolar anterior-posterior montage and referential (Cz) into the routine *EEG*. Then, the recordings were referenced to the common average value of the scalp *EEG* channels in the EEGLAB toolbox [30]. The parameters of the amplifier were a sensitivity at 10 μV/mm, a time constant equal to 0.3 s, and a high frequency filter at 70 Hz.

Written consent forms to participate in this study were obtained for all the participating subjects. The participants were asked to sit relaxed in an upright position with their eyes closed. Routine *EEG* recordings were sampled at 500 Hz, and the duration ranged from 11 to 17 min (13 min on average) for *AD* patients and from 20 to 23 min (21 min on average) for healthy subjects. In total, 179 min of *EEG* data from *AD* patients (116 min from mild *AD* and 63 min from moderate *AD*) and 187 min from healthy subjects were recorded.

### 2.2. Feature Extraction

**Figure 1:**
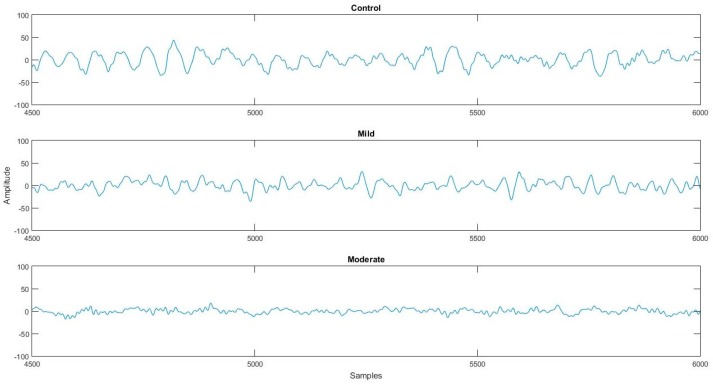
A segment of 3 s extracted from a 12-s epoch of O1 of three different subjects (control, mild Alzheimer’s Disease (AD) patient, and moderate AD patient).

The Nihon Kohden EEG 2100 device provides information about any possible artifacts during each *EEG* recording (electromyographic artifacts, blinking, and swallowing), which were marked and removed. The *EEG* signals are preprocessed using a high-pass *FIR* (Equiripple) digital filter with a cutoff frequency at 0.5 Hz to remove low frequencies around 0. Furthermore, a Butterworth notch filter was designed at 50 Hz to remove the 50 Hz power line noise interference from the *EEG* signals. The EEG recordings were filtered to the frequency range 0.5–60 Hz. In Figure 1, a plot of the O1 channel of three different subjects (control, mild AD patient, and moderate AD patient) is presented.

The *EEG* features were extracted from the filtered *EEG* segments and for each *EEG* rhythm. Specifically, the spectral and time-based features were extracted from the *EEG* segments of different window lengths for the entire spectrum, namely

1. Shannon entropy (ShanEN),
2. Multiscale entropy (MSE),
3. Mean,
4. Variance,
5. Standard deviation (STD),
6. Skewness,
7. Kurtosis, and
8. Interquartile Range (IQR).

Furthermore, the spectral features were calculated for each sub-band of interest, corresponding to the five *EEG* rhythms (*δ*, *θ*, *α*, *β*, and *γ*). Thus, five equiripple *FIR* filters were initially designed and applied to decompose the *EEG* segments to the specific sub-bands (0.5–4 Hz, 4–8 Hz, 8–13 Hz,13–30 Hz, and 30–60 Hz). Then, 6 spectral features were calculated for each band of the segment, namely

9. Energy of the *δ*, *θ*, *α*, *β*, and *γ* bands,
10. Relative band power (RBP) of the *δ*, *θ*, *α*, *β*, and *γ* bands,
11. Approximate entropy (ApEN) of the *δ*, *θ*, *α*, *β*, and *γ* bands,
12. Permutation entropy (PermEN) of the *δ*, *θ*, *α*, *β*, and *γ* bands,
13. Tsallis entropy (TsalEN) of the *δ*, *θ*, *α*, *β*, and *γ* bands,
14. Sample entropy (SamplEN) of the *δ*, *θ*, *α*, *β*, and *γ* bands,

The feature vector of 38 features (8 time-based and 6 × 5 spectral features) for each EEG channel (total 19), including the class attribute (thus, 38 × 19 + 1), was used to train a Random Forests [31] classifier. All calculations are implemented in MATLAB environment.

### 2.3. Classification

In order to find the optimal classification performance, a variety of classifiers (MultiLayer Perceptron, k-Nearest Neighbor, Support Vector Machines, Naïve Bayes, and Decision Trees [32]) were evaluated for whole-brain dynamics. In Table 2, the classification accuracy of the classifiers is presented. The Random Forests obtained the best classification results.

The Random Forests constructed multiple decorrelated decision trees using the bagging method. The decision trees were grown in binary partitioning, utilizing randomly selected features at each node to determine the split. Each decision tree was responsible for its own prediction, and in the end, they voted for the most popular class [31]. In the experiments, 100 decision trees were selected and the 10-fold cross-validation technique was employed.

## 3. Results

To evaluate the *EEG* window length and the proposed methodology, 6 classification problems are created. In the first problem, the group of 10 healthy subjects forms the class “controls” (CN), whereas the *EEG* features of all of the 14 *AD* patients are merged and forms the class “Alzheimer’s” (*AD*), resulting in the problem CN/AD. In the second problem (CN/mild/moderate), the *AD* group is further divided into the “mild” and “moderate” classes, corresponding to the groups of patients with mild *AD* (8 patients) and moderate *AD* (6 patients), respectively. The third problem is a 2-class problem between the controls and mild *AD* patients (CN/mild), whereas the forth problem consists of *EEG* features of the controls and moderate *AD* patients (CN/moderate). The fifth problem is a classification between two groups. The first group includes the moderate *AD* patients, and the second group consists of *EEG* data from the controls and patients with mild *AD* (CN-mild/moderate). Finally, the sixth problem corresponds to the classification among mild and moderate *AD* patients (mild/moderate).

The classifier’s performance is evaluated with Accuracy, Precision, F1-score, and kappa statistics. The accuracy of the classification shows the ability of the classifier to differentiate *AD* subjects from healthy subjects, healthy subjects from *AD* stages, and mild *AD* patients from moderate *AD* patients. The precision of the classification between *AD* patients and healthy subjects examines whether the correctly classified instances of *AD* patients are actual *AD* patients and whether the rest are healthy subjects incorrectly labeled as *AD*. On the other hand, the F1-score expresses the average of the precision and recall, wherein the recall shows whether the instances that should have been classified as *AD* are actually labeled as *AD* patients. The Kappa statistic evaluates the correctly classified instances and those that have been classified randomly owing to uncertainty [33]. The results for the six classification problems for 8 different window lengths (ranging from 5 to 12 s) are depicted in Table 3. For the 3-class problem (CN/mild/moderate), the average values are presented.

The best window length is 12 s for all classification problems with the classification accuracy ranging from 88.79% to 96.76% for the CN/mild/moderate and CN/moderate problems. The CN-mild/moderate problem indicates the second highest value of accuracy (94.99%), followed by CN/AD (91.80%), CN/mild (91.77%), and mild/moderate (91.71%). On the other hand, the worst classification results are obtained for epochs of 5 s. Likewise, CN/moderate shows the highest accuracy (94.68%), followed by the CN-mild/moderate (92.59%), mild/moderate (87.63%), CN/AD (86.98%), CN/mild (86.60%), and the 3-class problem CN/mild/moderate that succeeded the worst accuracy (82.34%). The classification accuracies for epochs of 6, 7, 8, 9, 10, and 11 s are gradually increased.

**Figure 2:**
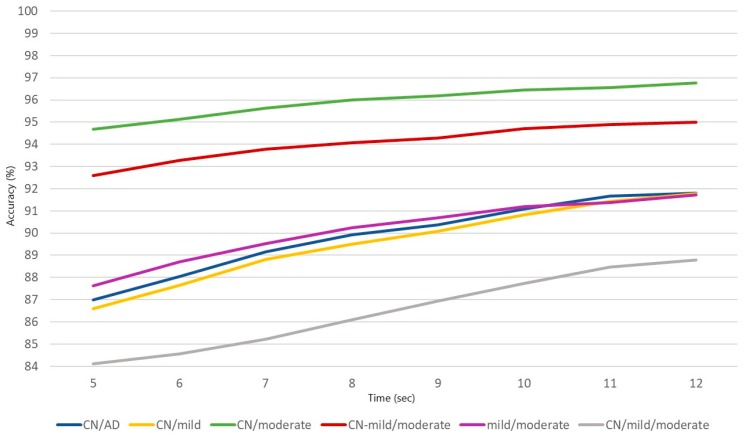
The results in terms of the classification accuracy for the six classification problems over 8 window lengths. (blue: CN/AD, yellow: CN/mild, green: CN/moderate, red: CN-mild/moderate, purple: mild/moderate, grey: CN/mild/moderate).

In Figure 2, a visualization of the obtained accuracy for each classification problem over different window lengths is presented.

Τhe rest of the analysis is conducted solely for the 12-s window length, which is the best classification window length according to the analysis. Table 4 presents the classification results (Accuracy, Precision, F1-score, and kappa statistics) as obtained for the best window length.

The best classification accuracy (96.76%), which also shows the highest kappa statistic (0.9069) and F1-score = 0.9277, is obtained for the 2-class problem CN/moderate, followed by the CN-mild/moderate (94.99%) with a kappa statistic of 0.8079 and an F1-score = 0.8372 and the CN-AD (91.80%) with a kappa statistic of 0.8340 and an F1-score = 0.9077. The 3-class problem CN-mild-moderate indicates the worse classification accuracy (88.79%) with a kappa of 0.8860 and an F1-score = 0.8474. The discrimination between the controls from mild Alzheimer’s (CN-mild) and between mild *AD* from moderate *AD* (mild-moderate) presents almost the same classification accuracy (91.77% and 91.71%, respectively) with the kappa statistics being 0.8132 and 0.8194, respectively, and the F1-scores equal to 0.8739 and 0.8837, respectively.

Furthermore, since the examination of different cortical regions is significant in *AD*, the electrodes are grouped in 5 groups, as proposed in previous studies [8,9] in order to capture the differences in the brain activities among subject groups in different brain regions. Thus, the 6 classification problems are also examined for epochs of 12 s for the anterior (Fp1, F3, Fz, Fp2, and F4), central (C3, Cz, and C4), left temporal (F7, T3, and T5), right temporal (F8, T4, and T6), and posterior (O1, O2, P3, Pz, and P4) clusters. The results are presented in Table 5. For the 3-class problem (CN/mild/moderate), the average values are presented.

A discrimination among the healthy subjects and Moderate *AD* patients (CN/moderate) indicates the best classification accuracy for all electrode clusters, ranging from 96.39% to 97.72% with kappa statistics from 0.8957 to 0.9338 and F1-scores from 0.9188 to 0.9469 for the anterior cluster, the right side of the temporal region, the left side of the temporal region, the central region, and the posterior region.

For the 2-class problem “CN-mild/moderate”, the central region shows the best classification results (ACC = 97.19%, kappa = 0.8796, and F1-score = 0.9163), followed by the posterior region (ACC = 96.95%, kappa = 0.8492, and F1-score = 0.9425), the left side of the temporal region (ACC = 95.71%, kappa = 0.8348, and F1-score = 0.8599), the right side of the temporal region (ACC = 95.23%, kappa = 0.8156, and F1-score = 0.9480), and the anterior cluster (ACC = 94.37%, kappa = 0.7833, and F1-score = 0.8161). For the 2-class problem “mild-moderate”, the best classification accuracy is 96.24% (kappa = 0.921 and F1-score = 0.9518) for the central cluster, followed by 94.66% (kappa = 0.8828 and F1-score = 0.9239) for the posterior cluster, 94.28% (kappa = 0.8778 and F1-score = 0.9234) for the temporal/left, 92.57% (kappa = 0.8339 and F1-score = 0.8884) for the temporal/right, and the worse accuracy 90.03% (kappa = 0.7883 and F1-score = 0.8610) for the anterior cluster.

For the classification problem “CN/mild”, the highest accuracy is 94.87% for the central cluster (kappa = 0.8807 and F1-score = 0.9179), followed by 93.55% (kappa = 0.8566 and F1-score = 0.9055) for the posterior cluster, 92.18% (kappa = 0.8186 and F1-score = 0.8754) for the temporal/left, 91.02% (kappa = 0.8065 and F1-score = 0.8769), and 90.84% (kappa = 0.7894 and F1-score = 0.8561) for both the temporal/right and anterior clusters.

**Figure 3:**
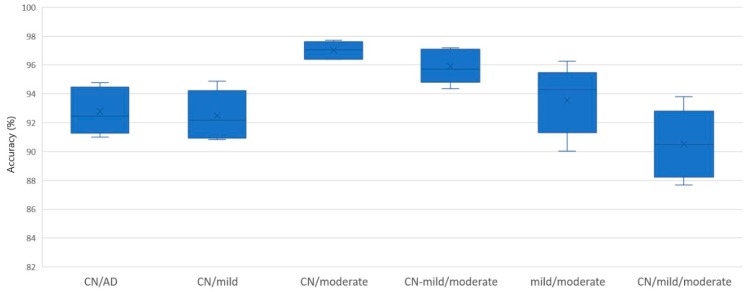
Boxplot of the accuracy results from all electrode clusters for each classification problem: The abbreviation CN stands for Controls and AD stands for Alzheimer’s Disease.

**Figure 4:**
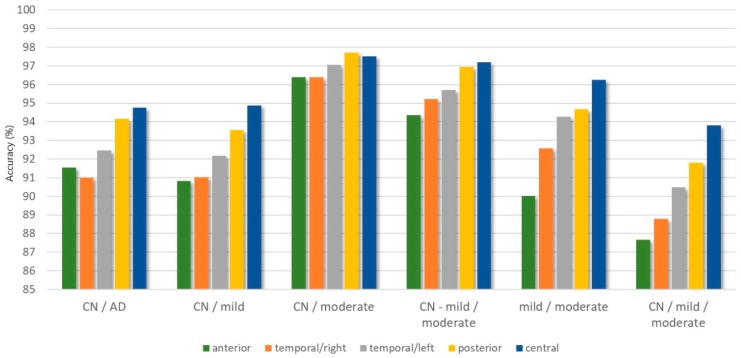
The distribution of the accuracy results in each cluster for the 5 classification problems: The abbreviation CN stands for Controls and AD stands for Alzheimer’s Disease.

The classification of Alzheimer’s concerning controls group (CN/AD) presents good classification results with accuracies ranging from 90.99% to 94.76% (temporal/right, anterior, temporal/left, posterior, and central), with kappa statistics from 0.8194 to 0.8936, and with F1-scores from 0.9148 to 0.9534. The worst classification performance is obtained for the 3-class problem (CN/mild/moderate) with an accuracy ranging from 87.67% to 93.80%, with kappa from 0.7861 to 0.8930, and with an F1-score from 0.8041 to 0.9051 for the anterior cluster, the right side of the temporal region, the left side of the temporal region, the posterior region, and the central region. A visualization of the obtained accuracy range for each classification problem is depicted in Figure 3. Figure 4 represents the classification accuracy in each cluster for each classification problem.

## 4. Discussion

In this study, a methodology for the detection of *AD*-related dynamics from the whole brain and from specific brain regions of interest was presented. The statistical and spectral features were calculated from the *EEG* segments of different lengths acquired from 14 patients with *AD* and 10 healthy subjects, which were used to train and test a Random Forests classifier. Six different classification problems were conducted for the evaluation of the proposed method.

The proposed methodology showed significant results in the discrimination between healthy elderly and *AD*-related patient groups and in the characterization of the disease (mild/moderate). With regard to the window length, the results showed a high classification accuracy as the length of the window was gradually increasing, and the best classification results were obtained for epochs of 12 s.

Furthermore, in this study, the brain asymmetry was examined since it was highly related to EEG information processing [34,35]. Generally, healthy elderly individuals showed a cortical atrophy which was predominantly affected by age and gradually resulted in *MCI* without significant functional alterations. Brain asymmetry in healthy individuals was present mainly in the right temporal lobe due to cortical thinning, and higher dynamics were shown. On the other hand, in *AD* patients, diffuse cortical atrophy, brain disfunction, and lower dynamics over the cerebral cortex were shown. The symptoms of patients with *AD* were due to pathological alterations in many regions of the cerebral cortex and became more severe as the disease progressed. The hippocampus was predominantly affected by *AD*, and hippocampal asymmetry was significantly reduced in *AD* patients. Also, functional magnetic resonance imaging (fMRI) studies [36] have shown additional atrophy in *AD* patients with *AD* in the medial temporal cortex, and it was evidence that the degree of brain asymmetry progressively decreased in *AD* patients [37]. The obtained results were consistent with the literature findings regarding functional abnormalities in *AD* patients compared to healthy, age-matched individuals. The results of the study indicated that *AD* was diagnosed better from *EEG* signals at the central and occipitoparietal regions and the left side of the temporal lobe than at the frontal area and at the right side of the temporal lobe. *AD*-related brain dynamics were discriminated from the ones acquired from healthy subjects better at the central and posterior regions for all classification problems (CN/mild, CN/moderate, CN/AD, CN-mild/moderate, and CN/mild/moderate) and the 2-class disease severity (mild-moderate). This outcome is in line with literature that suggests that the occipitoparietal area [1,29,38] and the left side of the brain [11,12] are more affected in *AD* than the frontal area and right hemisphere.

Also, a classification between healthy elderly subjects and dementia patients with moderate *AD* (CN-mild/moderate and CN/moderate) showed the best classification accuracy for a whole-brain classification and for each cluster separately. Undoubtedly, it was easier for the classifier to capture *EEG* changes between healthy elderly and *AD* patients with more severe disease progress, than between healthy individuals and mild *AD* patients, who showed less cognitive decline. Furthermore, the most challenging classification problem was the 3-class problem (CN/mild/moderate), which presented the worse performance in both the entire-brain classification and for each cluster. The low accuracy of this problem is mainly attributed to the misclassification of the mild *AD* group as the control group.

Most of previous studies [19,20,26] dealt with healthy elderly subjects, patients with *AD*, and patients with *MCI*, which is a prodromal stage of *AD*, not a category [19]. In this study, *MCI* patients were not included in the analysis. Therefore, it was not straightforward to compare the results of this study with previous reports related to *MCI*, and so, these studies were excluded from the comparison. The proposed method with statistical, spectral, and nonlinear features and Random Forests outperformed in the classification accuracy of a previous study [10] for all of the four binary classification problems (CN/AD, CN/mild, CN/moderate, and mild/moderate). Falk et al. [10] proposed a method wherein the Hilbert–Huang Transform was used to decompose *EEG* signals in 5 frequency bands, and then, the percentage modulation energy (PME) was extracted for each *EEG* rhythm. Support Vector Machines (SVM) were trained and tested with PME and obtained a 90.60% classification accuracy for the CN/AD problem. For the same classification problem, a Linear Discriminant Analysis classifier in a study [13] indicated a 90% accuracy with a maximum detrended cross-correlation coefficient when the C3-P3 channels were used as the input.

High levels of accuracy above 96% were obtained in References [11,12,14]. Kulkarni et al. [12] extracted wavelet, spectral, and complexity features from 50 AD patients and 50 healthy, age-matched subjects. The feature vector of the complexity features with SVM obtained a classification accuracy of 96% for the discrimination of *AD* patients from the controls (AD-CN); however, the *MMSE* score was not reported. Also, in Reference [14], the authors proposed a brain functional network construction method based on the calculation of multiscale entropy and evaluated several classifiers. The classification accuracy for the CN/AD problem with the k-Nearest Neighbor was above 96%. Nevertheless, the *MMSE* score of the *AD* subjects included in this study ranged from moderate *AD* to *MCI*. (*MMSE* score = 21.3 ± 5.8). Therefore, since, in our study, no *MCI* patients were included, a comparison with Reference [14] was not straightforward.

Another *AD* detection method was proposed in Reference [11], in which the proposed Multivariate Multiscale Weighted Permutation Entropy method with *ROC* curves achieved a 96.70% accuracy in the right frontal to the left occipitoparietal regions. However, the *MMSE* score of *AD* patients in this study ranged from 12–15, indicating a moderate *AD* stage. Thus, it was feasible to compare the abovementioned classification with the results of the “CN/moderate” problem of the proposed methodology, which showed a slightly better classification accuracy.

Simons and Abasolo [15] proposed a distance-based Lempel–Ziv complexity (dLZC) method to characterize the changes between pairs of electrodes and succeeded with a 78.25% accuracy for the O1–O2 pair. A comparison of the proposed methodology with previous studies is presented in Table 6.

## 5. Conclusions

*AD* is a severe neurodegenerative disease, and we know little about the underlying mechanics of the disorder. Currently, the challenge in the field of *AD*-related *EEG* analysis is to accurately diagnose dementia as early as possible towards a more efficient and tailored treatment plan in order to delay the progression of the disease. Research studies focus on evaluating specific *EEG* markers that provide a highly accurate discrimination of *AD* patients that are on different medication in order to assist neurologists in the adjustment of intervention plans in clinical trials [39,40,41]. The proposed study is an extend of our previous work [42] and investigated the ability of several statistical and spectral features to accurately discriminate *AD* patients with mild or moderate *AD* from healthy, age-matched subjects. Despite the good classification performance, improvements need to be done concerning the statistical significance of the results. Feature selection methods and other classification algorithms need to be evaluated to sustain the quality of the classification performance. Furthermore, this study is evaluated on 24 subjects. In a forthcoming study, *EEG* recordings from more participants will be analyzed.

A limitation of the proposed methodology is that no additional EEG preprocessing for artifacts removal was employed. This is mainly because, in the current study, the *EEG* signals were obtained so as to minimize *EMG* or other types of interferences. However, it is possible that an EEG recording may be contaminated with artifacts, and thus, methods detecting *EOG* and *EMG* artifacts are considered necessary in a robust and concise methodology. Future work will include the employment of *EEG* preprocessing techniques in order to detect and remove *EMG* or other types of artifacts.

## Figures and Tables

**Table 1:** The descriptions of participants’ characteristics.

	Moderate AD	Mild AD	Controls
Age	62.5 (61.25–68.25)	73.5 (68.5–77.25)	67 (62.25–72)
Gender (m:f)	3:3	3:5	7:3
Education level (P:S:H)	3:3	4:2:2	3:3:4
MMSE	15 (14–16)	21 (20–22)	30
Disease duration (months)	32 (24–36)	22 (19.5–24)	-

* m: male, f: female, P: primary education (6 years), S: secondary education (12 years), H: higher education (>12 years).

**Table 2:** The classification performance of MultiLayer Perceptron (MLP), k-Nearest Neighbor (KNN), Support Vector Machines (SVM), Naïve Bayes (NB), and Decision Trees (DT) for 12-s epochs in terms of accuracy.

Classification Problem	MLP	KNN	SVM	NB	DT	RF
CN/AD	86.11	80.98	77.23	66.20	83.29	**91.80**
CN/mild	89.02	85.16	77.11	54.80	85.76	**91.77**
CN/moderate	95.23	93.62	91.09	81.93	94.48	**96.76**
CN-mild/moderate	94.20	91.39	88.86	80.05	92.15	**94.99**
mild/moderate	90.17	87.48	79.15	70.03	86.30	**91.71**
CN/mild/moderate	80.71	74.88	66.59	46.74	77.55	**88.79**

**Table 3:** The classification results in terms of the Accuracy (ACC) for each classification problem for 8 window lengths.

Classification Problem	5 s	6 s	7 s	8 s	9 s	10 s	11 s	12 s
CN/AD	86.98	88.04	89.15	89.93	90.37	91.09	91.66	**91.80**
CN/mild	86.60	87.65	88.81	89.50	90.09	90.81	91.43	**91.77**
CN/moderate	94.68	95.13	95.64	95.99	96.18	96.46	96.56	**96.76**
CN-mild/moderate	92.59	93.27	93.78	94.06	94.29	94.70	94.88	**94.99**
mild/moderate	87.63	88.70	89.52	90.25	90.69	91.19	91.38	**91.71**
CN/mild/moderate	82.34	83.73	85.23	86.10	86.93	87.72	88.47	**88.79**

**Table 4:** The classification results in terms of the Accuracy (ACC), Precision, F1-Score, and Kappa Statistics for a 12-s window length.

Classification Problem	ACC (%)	Precision (%)	F1-score	Kappa
CN/AD	91.80	93.35	0.9077	0.8340
CN/mild	91.77	93.11	0.8739	0.8132
CN/moderate	96.76	97.78	0.9277	0.9069
CN-mild/moderate	94.99	92.24	0.8372	0.8079
mild/moderate	91.71	91.42	0.8837	0.8194
CN/mild/moderate	88.79	88.83	0.8474	0.8860

CN: Controls, AD: Alzheimer’s Disease.

**Table 5:** The classification results in terms of the Accuracy (ACC), Precision, F1-score, and Kappa statistics for the anterior (Fp1, F3, Fz, Fp2, and F4), central (C3, Cz, and C4), left/temporal (F7, T3, and T5), right/temporal (F8, T4, and T6), and posterior (O1, O2, P3, Pz, and P4) clusters. For the analysis, the electroencephalographic (EEG) signals were segmented in epochs of 12 nonoverlapping seconds.

	Classification Problem	ACC (%)	Precision (%)	F1-score	Kappa
*anterior*	CN/AD	91.53	90.32	0.9244	0.8283
	CN/mild	90.84	92.47	0.8561	0.7894
	CN/moderate	96.39	97.70	0.9188	0.8957
	CN-mild/moderate	94.37	90.78	0.8161	0.7833
	mild/moderate	90.03	89.48	0.8610	0.7835
	CN/mild/moderate	87.67	87.31	0.8041	0.7861
*central*	CN/AD	94.76	94.00	0.9534	0.8936
	CN/mild	94.87	96.44	0.9179	0.8807
	CN/moderate	97.51	97.68	0.9469	0.9307
	CN-mild/moderate	97.19	96.40	0.9163	0.8796
	mild/moderate	96.24	96.44	0.9518	0.9210
	CN/mild/moderate	93.80	94.43	0.9051	0.8930
*temporal/left*	CN/AD	92.45	91.98	0.9337	0.8462
	CN/mild	92.18	91.53	0.8754	0.8186
	CN/moderate	97.05	99.11	0.9319	0.9131
	CN-mild/moderate	95.71	94.49	0.8599	0.8348
	mild/moderate	94.28	93.78	0.9234	0.8778
	CN/mild/moderate	90.49	90.73	0.8528	0.8339
*temporal/right*	CN/AD	90.99	88.94	0.9148	0.8194
	CN/mild	91.02	92.12	0.8769	0.8065
	CN/moderate	96.40	97.95	0.9232	0.8997
	CN-mild/moderate	95.23	94.80	0.8434	0.8156
	mild/moderate	92.57	92.93	0.8884	0.8329
	CN/mild/moderate	88.78	89.83	0.8488	0.8112
*posterior*	CN/AD	94.17	93.90	0.9468	0.8823
	CN/mild	93.55	93.25	0.9055	0.8566
	CN/moderate	97.72	98.04	0.9485	0.9338
	CN-mild/moderate	96.95	94.20	0.9425	0.8492
	mild/moderate	94.66	93.29	0.9239	0.8828
	CN/mild/moderate	91.80	91.57	0.8981	0.8600

**Table 6:** A comparison of the performances of the various methods proposed in the literature related to Alzheimer’s Disease.

Authors	No. of Subjects	Window Length	MMSE Range	Method	Classification Problem	ACC
Falk et al. [10]	11 CN/11 mild/10 moderate	5 s	CN: 26.6 ± 2.7 mild: 18.5 ± 4.7 mod: 14.8 ± 3.9	HHT, Amplitude modulation analysis, SVM	CN/AD CN/mild CN/mod mild/mod	90.60% 74.10% 71.40% 53.80%
Deng et al. [11]	14 CN/14 AD	8 s	CN: 28–30 AD: 12–15	Multivariate Multiscale Weighted Permutation Entropy, ROC analysis	CN/AD	96.70%
Kulkarni et al. [12]	50 CN/50 AD	~5 s		Spectral entropy, Spectral centroid, Spectral roll-off, Zero Crossing Rate, SVM	CN/AD	96.00%
Chen et al. [13]	15 CN/15 AD	8 s	CN: 28.1–30 AD: 12.5–15.7	Detrended Fluctuation Analysis, Cross-correlation coefficient, LDA	CN/AD	90.00% (only C3–P3)
Song et al. [14]	15 CN/15 AD	8 s	CN: 27.1 ± 1.3 AD: 21.3 ± 5.8	Brain Functional Connectivity Analysis, weighted-permutation entropy, KNN	CN/AD	96.63%
Simons and Abasolo [15]	11 CN/11 AD	5 s	CN: 30 AD: 13.1 ± 5.9	Distance-based Lempel Ziv Complexity	CN/AD	78.25% (only O1–O2)
This study	10 CN/14 AD	12 s	CN: 30 mild: 21 ± 1.3 mod: 15 ± 1.6	moments, STD, IQR, Energy, RBP, ShanEN, ApEN, TsalEN, PermEN, MSE, SamplEN, Random Forests	CN/AD CN/mild CN/mod mild/mod CN-mild/mod CN/mild/mod	91.80% 91.77% 96.76% 91.71% 94.99% 88.79%

CN: Controls, AD: Alzheimer’s Disease, mod: moderate AD, HHT: Hilbert–Huang Transform, SVM: Support Vector Machines, LDA: Linear Discriminant Analysis, KNN: k-Nearest Neighbor, STD: Standard Deviation, IQR: Interquartile range, RBP: Relative Band Power, ShanEn: Shannon Entropy, ApEN: Approximate Entropy, TsalEN: Tsallis Entropy, PermEn: Permutation Entropy, MSEL Multiscale Entropy, SamplEN: Sample Entropy.
